# Episódios de Alta Frequência Atrial e sua Associação com Eventos Isquêmicos Cerebrais em Pacientes Chagásicos

**DOI:** 10.36660/abc.20190647

**Published:** 2020-12-01

**Authors:** Emanoela Lima Freitas, Elieusa e Silva Sampaio, Márcia Maria Carneiro Oliveira, Lucas Hollanda Oliveira, Marcos Sergio da Silva Guimarães, Jussara de Oliveira Pinheiro, Luís Pereira de Magalhães, Guisela Steffen Bonadie Albuquerque, Cristiano Macedo, Roque Aras

**Affiliations:** 1 Hospital Cardio Pulmonar SalvadorBA Brasil Hospital Cardio Pulmonar , Salvador , BA - Brasil; 2 Universidade Federal da Bahia SalvadorBA Brasil Universidade Federal da Bahia – Enfermagem, Salvador , BA - Brasil; 3 Escola de Enfermagem SalvadorBA Brasil Escola de Enfermagem , Salvador , BA - Brasil; 4 Hospital Ana Nery SalvadorBA Brasil Hospital Ana Nery , Salvador , BA - Brasil; 5 Hospital Universitário Professor Edgard Santos SalvadorBA Brasil Hospital Universitário Professor Edgard Santos , Salvador , BA - Brasil; 6 Universidade Federal da Bahia Faculdade de Medicina de Bahia SalvadorBA Brasil Universidade Federal da Bahia Faculdade de Medicina de Bahia , Salvador , BA – Brasil

**Keywords:** Doença de Chagas/complicações, Flutter Atrial, Infarto Cerebral, Isquemia Encefálica, Marca-Passo Artificial, Tomografia Computadorizada/métodos

## Abstract

**Fundamento:**

Episódios de alta frequência atrial (EAFAs) estão associados a um risco elevado de eventos isquêmicos cerebrais, porém não existem estudos relacionados com a presença de EAFAs e eventos isquêmicos cerebrais em pacientes chagásicos.

**Objetivo:**

Investigar a associação entre a presença de EAFAs ≥ 6 minutos e eventos isquêmicos cerebrais em pacientes chagásicos.

**Métodos:**

Estudo de coorte com pacientes chagásicos, portadores de dispositivos cardíacos eletrônicos implantáveis (DCEIs), acompanhados no ambulatório de arritmias de um hospital universitário, na cidade de Salvador/BA, entre maio de 2016 e junho de 2017. Pacientes com diagnóstico de flutter atrial/fibrilação atrial, com DCEI unicameral e em uso de anticoagulação oral foram excluídos. Foram considerados EAFAs com frequência atrial ≥ 190 batimentos por minuto e duração ≥ 6 minutos (min), e os eventos isquêmicos cerebrais foram identificados por meio de tomografia computadorizada (TC) de crânio.

**Resultados:**

Os 67 participantes da pesquisa (67,2% do sexo feminino, com idade média de 63,6 ± 9,2 anos) foram acompanhados por 98 ± 28,8 dias e 11,9% dos pacientes apresentaram EAFAs ≥ 6 minutos. A TC de crânio evidenciou eventos isquêmicos cerebrais silenciosos em 16,4% dos pacientes, sendo que, destes, 63,6% haviam apresentado os EAFAs ≥ 6 minutos na análise dos DCEIs. A idade avançada (OR 1,12 [IC 95% 1,03-1,21; p=0,009) e a presença de EAFAs ≥ 6 minutos (OR 96,2 [IC 95% 9,4-987,4; p<0,001]) foram preditores independentes para eventos isquêmicos.

**Conclusão:**

EAFAs detectados por DCEIs estavam associados à presença de eventos isquêmicos cerebrais silenciosos em pacientes chagásicos. (Arq Bras Cardiol. 2020; [online].ahead print, PP.0-0)

## Introdução

A fibrilação atrial (FA) aumenta o risco de acidente vascular cerebral (AVC) isquêmico em cerca de 5 a 6 vezes, independentemente de outros fatores de risco. ^1^ Nos últimos anos, tem crescido o interesse em detectar FA em uma fase mais precoce, antes da identificação clínica, com detecção principalmente por meio de marca-passo/desfibrilador cardíaco implantável (CDI) e que precedem a primeira manifestação da doença. ^2 , 3^ São os episódios de alta frequência atriais (EAFAs), que correspondem à ocorrência de arritmias atriais como fibrilação e *flutter* atrial, e são caracterizados por apresentar uma frequência atrial ≥ 190 batimentos por minuto (bpm) ^4^ ou ≥250 bpm, ^3^ com duração ≥ 5 a 6 minutos (min). São episódios assintomáticos e detectados apenas através de monitoramento contínuo, e denominados também “FA subclínica”. ^4^

EAFAs estão associados a um risco elevado de AVC ^5^ e a tendência é que esses episódios tenham o mesmo prognóstico adverso que a FA clínica; no entanto, a duração, a frequência ou a carga diária exata desses episódios no risco de AVC ainda são desconhecidas; assim, o limiar de EAFAs que justifica a anticoagulação oral ainda não está claro. ^6^

A incidência de EAFAs é de 30% a 70%, dependendo do perfil clínico da população estudada e dos algoritmos de detecção utilizados em cada protocolo de estudo. ^7^ Ao excluir pacientes com história de FA e em uso de anticoagulação oral, esse número cai para cerca de 30%. ^8 - 10^

Entretanto, em algumas populações específicas e vulneráveis às complicações tromboembólicas, como em pacientes portadores de doença de Chagas (DC), não existem dados relacionados à investigação da presença desses episódios e sua incidência.

Aproximadamente 30% dos pacientes com DC desenvolvem as alterações cardíacas, e mais de 10%, alterações neurológicas. ^11^ As complicações da doença cardíaca são principalmente em decorrência de arritmias e insuficiência cardíaca, responsáveis por mais de 35% dos óbitos. ^12^ Na DC, a FA é a arritmia supraventricular mais frequente, sendo encontrada em 4 a 12% dos casos, ^13^ sendo uma das principais causas dos eventos embólicos cerebrais, ^14^ cuja incidência chega a atingir 3% da população chagásica. ^15^

Investigar a presença dos EAFAs e sua associação com AVC em pacientes com DC pode permitir a inclusão desses pacientes na utilização de anticoagulantes orais. O objetivo foi investigar a associação entre a presença de EAFAs ≥ 6 minutos e eventos isquêmicos cerebrais em pacientes chagásicos.

## Métodos

### Desenho do Estudo e População

Trata-se de um estudo observacional, do tipo coorte prospectiva. Foram incluídos 77 pacientes, de ambos os sexos, com idade ≥ 18 anos, acompanhados no ambulatório de arritmias de um hospital universitário referência em Cardiologia, na cidade de Salvador/BA, entre maio de 2016 e junho de 2017. Os pacientes eram portadores de doença de Chagas e DCEIs (marca-passo, cardiodesfibrilador implantável ou dispositivos de terapia de ressincronização cardíaca) capazes de monitorar a atividade atrial. Foram excluídos os pacientes com diagnóstico de fibrilação atrial/ *flutter* atrial, portadores de DCEI unicameral, aqueles com indicação crônica de anticoagulação oral por qualquer razão ou que tinham contraindicação à realização de TC de crânio.

A pesquisa foi conduzida de acordo com os princípios da Declaração de Helsinque e aprovada pelo Comitê de Ética em Pesquisa do Hospital Universitário Professor Edgard Santos (UFBA-HUPES) (sob o número: 1.426.885, em 26/02/2016). O Termo de Consentimento foi obtido dos participantes.

Foram coletados dados sobre sexo, idade, etnia, comorbidades, tipo e indicação dos DCEIs, modo de estimulação dos DCEIs, caracterização da doença de Chagas, terapêutica medicamentosa, além de dados relacionados à radiografia de tórax, ecocardiograma transtorácico (ECO-TT) e eletrocardiograma (ECG) de longa duração – Holter 24 horas. A DC foi classificada, em cada paciente, conforme critérios do Consenso Brasileiro em Doença de Chagas.14 Os dados foram coletados a partir de entrevistas com os pacientes e prontuários.

Foi utilizado o escore de risco CHA _2_ DS _2_ -VASc – C: *congestive heart failure* or *left ventricular systolic dysfunction* (insuficiência cardíaca congestiva ou disfunção sistólica do ventrículo esquerdo) = 1 ponto, H: *hypertension* (hipertensão) = 1 ponto, A _2_: *age* (idade) ≥ 75 anos = 2 pontos, D: *diabetes mellitus* (diabetes melito) = 1 ponto, S _2_: *prior stroke or transient ischemic attack or thromboembolism* (AVC prévio ou ataque isquêmico transitório ou tromboembolismo) = 2 pontos, V: *vascular disease* ( *p. ex., peripheral artery disease, myocardial infarction, aortic plaque* ) (doença vascular [p. ex., doença arterial periférica, infarto do miocárdio, placa aórtica]) = 1 ponto, A: *age* (idade) 65 a 74 anos = 1 ponto e Sc: *sex category* (female sex) (gênero [sexo feminino]) = 1 ponto. ^16^

A classificação de risco de eventos cerebrovasculares, de acordo com o escore CHA2DS2-VASc é a seguinte: alto risco (2 pontos ou mais), risco intermediário (1 ponto) e baixo risco (0 ponto).

### Procedimentos do Estudo

Após assinatura do termo de consentimento informado, os pacientes foram submetidos à realização de ECG de 12 derivações, visando à confirmação da ausência de fibrilação atrial/ *flutter* atrial, além da descrição do ritmo cardíaco e distúrbios de condução intraventricular. Em seguida, os pacientes foram avaliados por um médico arritmologista e tiveram seus DCEIs ajustados para uma programação específica, visando à detecção e gravação das arritmias atriais.

Depois de um período de aproximadamente 3 meses após a programação, os pacientes retornaram ao ambulatório para análise dos dispositivos (leitura dos DCEIs), com o intuito de identificar e classificar a ocorrência das arritmias atriais, os EAFAs.

Durante o período entre a programação e a leitura dos DCEIs, os pacientes foram submetidos à realização da TC de crânio sem contraste, com o objetivo de identificar eventos isquêmicos cerebrais. Foram identificados como infarto cerebral silencioso aqueles pacientes que apresentavam alterações de infarto cerebral nos laudos das TCs e que não tinham alterações clínicas de eventos isquêmicos ou déficits neurológicos. As TCs foram realizadas e avaliadas pelo departamento de neurorradiologia do hospital. Esse exame foi realizado em aparelho da Toshiba Medical Systems Corporation, 1385 (Shimo Ishigami, Otawara-Shi, Tochigi, Japão).

### Programação dos DCEIs

A escolha do fabricante do dispositivo não influenciou na inclusão/exclusão do paciente. Foram incluídos dispositivos dos fabricantes Medtronic®, StJude Medical® e Biotronik®, cujos modelos estavam disponíveis para uso nessa população.

O dispositivo foi programado para identificar EAFAs com duração de pelo menos 190 bpm, com duração ≥ 6 minutos, reconhecido como um ponto de corte apropriado para selecionar EAFAs e descartar a contração atrial prematura ou falsos eventos ^17^ durante todo período de monitoramento. Essa duração foi escolhida por ser consistente com a metodologia de dois grandes estudos – *Asymptomatic Atrial Fibrillation and Stroke Evaluation in Pacemaker Patients and the Atrial Fibrillation Reduction Atrial Pacing Trial* (ASSERT) ^4^ e *The Relationship Between Daily Atrial Tachyarrhythmia Burden From Implantable Device Diagnostics and Stroke Risk* (TRENDS) ^3^ – que demonstraram a associação entre EAFAs, com duração de pelo menos 5 ou 6 minutos, e eventos isquêmicos cerebrais. O eletrograma de armazenamento foi ativado para confirmar a ocorrência dos EAFAs. Os pacientes tiveram a sensibilidade atrial programada para 0,1 a 0,5 milivolts (mV).

Todos os EAFAs detectados pelo DCEI com duração ≥ 6 minutos e com frequência ≥ 190 bpm foram documentados e enviados para avaliação cega do especialista (médico eletrofisiologista). A ativação de outros desencadeantes do armazenamento de eletrogramas foi deixada a critério do médico, mas não foi apoiada pelo protocolo do estudo.

Além da programação dos dispositivos, foram coletados dados relacionados com parâmetros de estimulação/detecção, percentuais de utilização atrial e ventricular, além de frequência cardíaca mínima e máxima.

### Acompanhamento

Após aproximadamente 3 meses desde a programação do DCEI, o paciente retornou para a consulta subsequente e, nesse momento, foram coletados os dados relacionados com a detecção dos EAFAs.

Os eletrogramas atriais correspondentes aos EAFAs detectados foram avaliados por médico eletrofisiologista cego aos resultados das TCs de crânio. Tais eletrogramas foram categorizados como detecções adequadas ou inadequadas. As detecções inadequadas (ruídos, detecção da onda *Farfield* ventricular ou sincronização ventriculoauricular repetitiva e não reentrante) ^17 , 18^ foram excluídas.

Foram definidas três categorias sem sobreposição da duração dos EAFAs: (1) sem EAFAs; (2) EAFAs com duração < 6 minutos; (3) EAFAs ≥ 6 minutos. A partir da detecção dos EAFAs com duração ≥ 6 minutos, foram definidos 4 subgrupos: (1) EAFAs entre 6 minutos e 29 minutos; (2) EAFAs entre 30 minutos e 5 horas e 59 minutos; (3) EAFAs entre 6 horas e 23 horas e 59 minutos e (4) EAFAs ≥ 24 horas. Esses cortes foram definidos de acordo com os valores preditivos positivos avaliados em uma análise do estudo *ASSERT* . ^19^

Para EAFAs ≥ 6 minutos e ≥ 190 batimentos/min, o valor preditivo positivo foi de 82,7%. O valor preditivo positivo aumentou para 93,2%, 96,7% e 98,2% quando a duração do limiar foi prolongada para 30 minutos, 6 horas e 24 horas, respectivamente. O aumento da frequência cardíaca limiar para 250 batimentos/min diminuiu as detecções de falso-positivo, mas em menor medida, e adicionou-se apenas marginalmente ao valor preditivo positivo quando foram utilizadas longas durações de limiar. ^19^

### Eventos Isquêmicos na Tomografia de Crânio

Todas as TCs foram realizadas e avaliadas pelo departamento de neurorradiologia do hospital onde o estudo foi realizado, com o objetivo de identificar áreas compatíveis com eventos isquêmicos (áreas isquêmicas, infartos lacunares, glioses localizadas ou áreas hipodensas). Para pacientes sem história clínica ou déficits neurológicos anteriores, os eventos isquêmicos foram considerados como silenciosos. As TCs de crânio foram realizadas em aparelho da Toshiba Medical Systems Corporation, 1385, Shimoishigami, Otawara-Shi, Tochigu, Japão.

### Análise Estatística

A análise estatística foi realizada utilizando o programa IBM SPSS, versão 21.0 (IBM, Armonk, New York). Os dados foram submetidos à análise estatística descritiva, por meio de medições de frequência (absolutas e relativas) para variáveis qualitativas. Para as variáveis quantitativas, utilizaram-se média e desvio-padrão ou mediana e intervalo interquartil, a depender da distribuição da variável, que foi testada por meio do teste de Shapiro-Wilk.

Para as variáveis categóricas, foi aplicado o teste χ ^2^ ou teste exato de Fisher. O teste Student t não pareado foi utilizado para as médias das idades dos pacientes com EAFAs ≥ 6 minutos e sem EAFAs ≥ 6 minutos e para a comparação das médias da FEVE, e o teste de Mann-Whitney foi empregado para comparação do implante do DCEI em meses.

Os resultados foram apresentados por meio de *odds ratio* (OR) e os seus respectivos intervalos de confiança de 95% (IC95%). Um valor de p < 0,05 foi considerado estatisticamente significante.

## Resultados

### Características da População e Detecção dos Eafas

Foram incluídos 77 pacientes na coorte – 10 foram excluídos, sendo que 7 apresentaram FA no momento da programação do dispositivo cardíaco, sendo iniciada anticoagulação oral (por definição do médico assistente do paciente); 2 foram a óbito antes da leitura do dispositivo e em 1 paciente foi identificado *farfield* ventricular durante leitura do DCEI. Ao final, 67 participantes cumpriram todas as etapas da pesquisa.

A média de idade foi de 63,6 ± 9,2 anos; todos os participantes estavam na fase crônica da DC, sendo que 89,6% deles haviam desenvolvido a forma cardíaca e 10,4% deles desenvolveram a forma cardiodigestiva da doença. Dentre as manifestações clínicas da forma cardíaca da DC, estavam presentes: síndrome arrítmica (100% dos pacientes), insuficiência cardíaca (IC) (38,8%) e síndrome tromboembólica (3%, correspondendo a 2 pacientes com AVC prévio). As manifestações clínicas relacionadas com a forma cardiodigestiva consistiram em ocorrência de megaesôfago chagásico em 7 pacientes.

Quanto aos dados relacionados com DCEIs, identificamos que 46,3% dos pacientes estavam em modo de estimulação DDD (estimulação de dupla câmara), seguido de 41,8% do modo DDD-R (DDD com resposta de frequência – R); a frequência cardíaca mínima foi de 61,8 ± 3,9 bpm, e máxima de 124,4 ± 5,5 bpm. O percentual de utilização atrial teve média de 52,8 ± 37,4% e o ventricular, 65,6 ± 42,5%.

A média de acompanhamento foi de 98 ± 28,8 dias e foram detectados EAFAs em 24 (35,8%) pacientes, com durações variadas. A incidência de EAFAs com duração ≥ 6 minutos ou “FA subclínica” foi de 11,9% (n = 08).

A mediana de tempo para atingir o primeiro EAFA foi de 26,2 dias (variando entre 0,08 e 83,25 dias), e a mediana da duração dos EAFAs foi de 135,4 minutos (variando entre 22,8 e 5.811,8 minutos).

As comparações das características demográficas e clínicas dos pacientes com EAFAs ≥ 6 minutos *versus* pacientes sem EAFAs ou com duração < 6 minutos são demonstradas na Tabela 1 .

**Tabela 1:** – Características demográficas e clínicas dos pacientes com EAFAs ≥ 6 minutos *versus* pacientes sem EAFAs ou com duração < 6 minutos

Características demográficas e clínicas	População total ( *n* = 67)	Com EAFAs ≥ 6 minutos ( *n* = 08)	Sem EAFAs ou < 6min (n = 59)	Valor de p
Idade (anos) – média ± DP	63,6 ± 9,2	69,9 ± 10,4	62,8 ± 8,8	0,040 ^†^
Sexo – n (%)				0,103
Feminino	45 (67,2)	3 (6,7)	42 (93,3)	
Masculino	22 (32,8)	5 (22,7)	17 (77,3)	
Etnia – n (%)				1,000
Branco	4 (6)	0 (0,0)	4 (100)	
Não branco	63 (94)	8 (12,7)	55 (87,3)	
Tipo de DCEI				1,000
Marca-passo	62 (92,5)	8 (12,9)	54 (87,1)	
CDI	5 (7,5)	0 (0,0)	5 (100)	
Indicação do DCEI – n (%)				1,000
BAV/BAVT	52 (77,6)	7 (13,5)	45 (86,5)	
DNS	10 (14,9)	1 (10,0)	9 (90)	
Prevenção secundária MSC	5 (7,5)	0(0,0)	5 (100)	
Implante do DCEI (meses) ^§^	108 (48-168)	144 (54-165)	96 (36-180)	0,757*
IC (NYHA)	26 (38,8)	4 (15,4)	22 (84,6)	0,701
Classe funcional da IC				1,000
I	7 (26,9)	1 (14,3)	6 (85,7)	
II	13 (50)	2 (15,4)	11 (84,5)	
III	6 (23,1)	1 (16,7)	5 (83,3)	
IAM – n (%)	4 (6)	8 (12,7)	4 (100)	1,000
HAS – n (%)	50 (74,6)	6 (12)	44 (88)	1,000
Diabetes – n (%)	6 (9)	1 (16,7)	5 (83,3)	0,549
Dislipidemia – n (%)	21 (31,3)	2 (9,5)	19 (90,5)	1,000
FEVE % – média ± DP	58,5 ± 14,1	58,1 ± 11	58,6 ± 14,5	0,495
AE ≥ 40 mm – n (%)	22 (32,8)	3 (13,6)	19 (86,4)	1,000
Escore CHA2DS2-VASc				0,346
Risco baixo	12 (17,9)	1 (8,3)	11 (91,7)	
Risco intermediário	37 (55,2)	3 (8,1)	34 (91,9)	
Risco alto	18 (26,9)	4 (22,2)	14 (77,9)	

Fonte: próprio autor Os dados são apresentados em média ± desvio-padrão ou o número de pacientes (%). §Dados apresentados em mediana e intervalo interquartil. Os valores de P foram calculados usando o teste qui-quadrado. *Mann-Whitney e o †teste t de Student, conforme apropriado. DCEI: dispositivo cardíaco eletrônico implantável; CDI: cardiodesfibrilador implantável; BAV/BAVT: bloqueio atrioventricular/bloqueio atrioventricular total; DNS: doença do nó sinusal; MSC: morte súbita cardíaca; IC: insuficiência cardíaca (NYHA: New York Heart Association); IAM: infarto agudo do miocárdio; HAS: hipertensão arterial sistêmica; FEVE: fração de ejeção do ventrículo esquerdo; AE: átrio esquerdo; CHA2DS2-VASc: escore de risco de eventos tromboembólicos (C: congestive heart failure or left ventricular systolic dysfunction (insuficiência cardíaca congestiva ou disfunção sistólica do ventrículo esquerdo) = 1 ponto, H: hypertension (hipertensão) = 1 ponto, A _2_: age (idade) ≥ 75 anos = 2 pontos, D: diabetes mellitus (diabetes melito) = 1 ponto, S _2_: prior stroke or transient ischemic attack or thromboembolism (AVC prévio ou ataque isquêmico transitório ou tromboembolismo) = 2 pontos, V: vascular disease (p. ex., peripheral artery disease, myocardial infarction, aortic plaque) (doença vascular [p. ex., doença arterial periférica, infarto do miocárdio, placa aórtica]) = 1 ponto, A: age (idade) 65 a 74 anos = 1 ponto e Sc: sex category (female sex) (gênero [sexo feminino]) = 1 ponto).

### Detecção dos Eventos Isquêmicos

Onze pacientes (16,4%) apresentaram evento isquêmico na TC de crânio e não tinham história de AVC prévio. Observou-se que 87,5% dos pacientes que tinham EAFAs ≥ 6 minutos, apresentaram eventos isquêmicos na TC de crânio. Na Tabela 2 , demonstram-se as características clínicas dos pacientes com e sem eventos isquêmicos.

**Tabela 2:** – Características clínicas dos pacientes com e sem eventos isquêmicos

Características clínicas	Eventos isquêmicos ( *n* = 11)	Sem eventos isquêmicos ( *n* = 56)	Valor de p
Idade (anos) – média ± DP	70,6 ± 10,9	62,2 ± 8,3	0,005 ^†^
Sexo – n (%)			0,483
Feminino	6 (13,3)	39 (86,7)	
Masculino	5 (22,7)	17 (77,3)	
IC	6 (23,1)	20 (76,9)	0,315
Classificação da IC – (NYHA)			0,843
I	1 (14,3)	6 (85,7)	
II	3 (23,1)	10 (76,9)	
III	2 (33,3)	4 (66,7)	
IAM – n (%)	1 (25)	3 (75)	0,521
HAS – n (%)	8 (16)	42 (84)	1,000
DM – n (%)	1 (16,7)	5 (83,3)	1,000
Dislipidemia – n (%)	5 (23,8)	16 (76,2)	0,301
FEVE % – média ± DP	57,2 ± 14	58,7 ± 14,2	0,553 ^†^
AE ≥ 40mm – n (%)	6 (27,3)	16 (72,7)	0,157
Escore CHA2DS2-VASc			0,050
Risco baixo	-	12 (100)	
Risco intermediário	5 (13,5)	32 (86,5)	
Risco alto	6 (33,3)	12 (66,7)	
EAFAs			< 0,001
Sem EAFAs	3 (7)	40 (93)	
EAFAs < 6 minutos	1 (6,3)	15 (93,8)	
EAFAs ≥ 6 minutos	7 (87,5)	1 (12,5)	

*Fonte: próprio autor Os dados são apresentados em média ± desvio-padrão ou o número de pacientes (%). §Dados apresentados em mediana e intervalo interquartil. Os valores de P foram calculados usando o teste do qui-quadrado e o †teste t de Student. (§ - não consta esse símbolo na tabela) EAFAs: episódios de alta frequência atrial; DCEI: dispositivo cardíaco eletrônico implantável; MP: marca-passo cardíaco; BAV/BAVT: bloqueio atrioventricular/bloqueio atrioventricular total; DNS: doença do nó sinusal; MSC: morte súbita cardíaca; IC: insuficiência cardíaca; NYHA: New York Heart Association; IAM: infarto agudo do miocárdio; HAS: hipertensão arterial sistêmica; DM: diabetes melito; FEVE: fração de ejeção do ventrículo esquerdo; AE: átrio esquerdo; CHA2DS2-VASc: escore de risco de eventos tromboembólicos (C: congestive heart failure or left ventricular systolic dysfunction (insuficiência cardíaca congestiva ou disfunção sistólica do ventrículo esquerdo) = 1 ponto, H: hypertension (hipertensão) = 1 ponto, A _2_: age (idade) ≥ 75 anos = 2 pontos, D: diabetes mellitus (diabetes melito) = 1 ponto, S _2_: prior stroke or transient ischemic attack or thromboembolism (AVC prévio ou ataque isquêmico transitório ou tromboembolismo) = 2 pontos, V: vascular disease (p. ex., peripheral artery disease, myocardial infarction, aortic plaque) (doença vascular [p. ex., doença arterial periférica, infarto do miocárdio, placa aórtica]) = 1 ponto, A: age (idade) 65 a 74 anos = 1 ponto e Sc: sex category (female sex) (gênero [sexo feminino]) = 1 ponto).*

Identificou-se que 45,5% dos pacientes com eventos isquêmicos apresentaram EAFAs com duração entre 30 minutos e 5 horas e 59 minutos, e o número médio de EAFAs ≥ 6 minutos foi de 3,88 ± 2,58, sendo que 50% dos pacientes tiveram entre 1 e 3 episódios.

Além de considerar o maior EAFA identificado pelo DCEI, foi mensurada também a carga diária total de EAFAs (o máximo de tempo em que o paciente permaneceu em “FA subclínica” em 24 horas) e sua possível associação com eventos isquêmicos. Evidenciou-se que o paciente chagásico com carga diária com duração ≥ 6 minutos tem mais chance de desenvolver eventos isquêmicos (OR: 46,67 [6,57 – 331,67; p <0,001]). A mediana da carga diária máxima que teve associação com a ocorrência de eventos isquêmicos foi de 4.554 segundos (75,9 minutos) (OR: 1,001; p < 0,026).

A Tabela 3 , mostra a descrição da carga diária de EAFAs em pacientes com e sem eventos isquêmicos.

**Tabela 3:** – Descrição da carga diária de EAFAs em pacientes com e sem eventos isquêmicos

Descrição da carga diária	Com eventos isquêmicos ( *n* = 11)	Sem eventos isquêmicos ( *n* = 56)	Valor de p
Carga diária de EAFAs – n (%)			< 0,001
Sem carga diária	3 (27,3)	40 (71,4)	
< 6 minutos	1 (9,1)	14 (25)	
≥ 6 minutos	7 (63,6)	2 (3,6)	

*Os dados são apresentados através do número de pacientes (%). O valor de p foi calculado por meio do teste exato de Fischer. EAFAs: episódios de alta frequência atrial.*

A idade avançada e a presença de EAFAs ≥ 6 minutos foram associadas à eventos isquêmicos, conforme demonstrado na Tabela 4 .

**Tabela 4:** – Fatores associados aos eventos isquêmicos

Fatores associados	OR	IC 95%	Valor de p
EAFAs ≥ 6 minutos	96,2	9,4 - 987,5	< 0,001
Idade avançada	1,12	1,03 - 1,21	0,009
IC	2,16	0,58 -7,98	0,241
Dislipidemia	2,08	0,56 – 7,80	0,270


*OR: odds ratio; IC95%: intervalo de confiança de 95%; Teste χ ^2^ ; EAFAs: episódios de alta frequência atrial; IC: insuficiência cardíaca; HAS: hipertensão arterial sistêmica.*

## Discussão

Houve uma associação de EAFAs ≥ 6 minutos com eventos isquêmicos silenciosos. O estudo de Benezet-Mazuecos et al. ^20^ evidenciou que eventos isquêmicos cerebrais silenciosos ocorrem mais nos pacientes com EAFAs (42%) do que naqueles sem EAFAs (19%), encontrando uma OR de 3,4. Benezet-Mazuecos et al. ^21^ também descreveram que os eventos isquêmicos cerebrais silenciosos ocorreram mais nos pacientes com EAFAs (32%) do naqueles sem EAFAs (13%), encontrando uma OR de 2,45. Além disso, breves episódios de FA subclínica (48 horas) documentados pelo monitoramento do Holter foram associados a um risco significativamente aumentado de eventos isquêmicos cerebrais silenciosos e AVC. ^22^

A incidência de EAFAs em pacientes sem história de FA é de cerca de 30%, em períodos de monitoramento variados. ^4 , 9 , 10^ Em nosso estudo, a incidência encontrada na população chagásica (também sem história de FA), em um período de monitoramento de cerca de 3 meses, foi de 11,9%. Tal achado é muito similar ao encontrado em um grande estudo de 2.580 pacientes não chagásicos e sem história de FA, em que os EAFAs ≥ 6 minutos foram encontrados em 35% dos pacientes em um seguimento médio de 2,5 anos, e em 10% dos pacientes nos primeiros 3 meses do estudo. ^4^ No entanto, é importante ressaltar que a mediana de tempo para detecção dos episódios no nosso estudo com pacientes chagásicos (26,2 dias) foi inferior a de outros estudos, como no *MOde Selection Trial* (MOST) – 100 dias ^2^ – e no *ASSERT* – 36 dias. ^4^ Além disso, o número médio de EAFAs no nosso estudo foi maior (3,9) que o do estudo ASSERT (2,0). ^4^

Neste estudo, todos os pacientes possuíam um DCEI dupla câmara; entretanto, não se encontrou associação entre o modo de estimulação dupla câmara e a presença ou ausência de EAFAs, e isso pode ser devido ao fato de que muitos episódios foram de curta duração. No entanto, é possível que em uma amostra maior de pacientes, uma redução dos EAFAs, com estimulação da dupla câmara possa ser detectada. O estudo MOST ^2^ também não evidenciou essa associação.

A detecção dos EAFAs foi maior em pacientes com idade avançada, do sexo feminino, de etnia negra, com passado de IAM, cuja indicação do implante do DCEI foi o BAV/BAVT; contudo, apenas a idade avançada foi associada ao evento isquêmico. O envelhecimento da população traz consigo o aumento da doença cardíaca subjacente, e a melhora das técnicas de diagnóstico tem evidenciado uma alta prevalência de FA em pessoas com mais de 70 anos de idade. ^23^ Isso também acontece com a população chagásica, ^24^ onde a prevalência de FA foi acentuadamente maior com a idade avançada. Estudos evidenciaram que EAFAs eram mais prevalentes na população idosa, mas não foi encontrado significância estatística nessa associação. ^4 , 20^

Além dos eventos isquêmicos silenciosos associados com EAFAs ≥ 6 minutos em pacientes mais velhos, também houve associação do escore CHA2DS2-VASC de risco elevado com eventos isquêmicos silenciosos, semelhantes a outros estudos. ^20 , 21 , 25^

Apesar de os EAFAs detectados por DCEIs estarem associados a um aumento de 2 a 2,5 vezes no risco de AVC, se comparados com indivíduos sem EAFAs, ^3 , 4^ o risco absoluto de AVC entre esses pacientes é menor do que entre os portadores de FA clínica detectada. ^26^

Os estudos de detecção da “FA subclínica” através de DCEIs têm tentado identificar o limite de tempo ideal (considerando o maior episódio ou a carga diária) e suas consequências clínicas, tais como eventos tromboembólicos. As durações dos limiares descritos nos estudos têm sido altamente variáveis: 5 minutos, ^2 , 3^ sugerindo aumento de 2,8 no risco de AVC ou óbito; 6 minutos, ^4^ com aumento de 2,5 maior risco para tromboembolismo; 24 horas, ^8^ com aumento de 3,1 com maior risco para eventos tromboembólicos. Da mesma forma, a carga diária de 3,8 ^27^ e 5,5 horas ^3^ também tem sido associada a um aumento significativo do risco de AVC (aumento de 9 e 2 vezes, respectivamente).

No nosso estudo, a carga diária dos EAFAs com duração ≥ 6 minutos sugere maior chance de o paciente chagásico desenvolver evento isquêmico silencioso, um risco mais elevado do que o descrito por outros estudos. Em pacientes com DCEIs, ^28^ evidenciou-se uma OR de 2,11 para a ocorrência de eventos isquêmicos nos pacientes que tiveram pelo menos 1 dia com no mínimo 1 hora da carga de “FA subclínica” (IC 95%: 1,22-3,64, p = 0,008). Outro estudo ^20^ descreve que a carga detectada pelos DCEIs demonstrou-se significativamente associada a eventos cerebrais isquêmicos silenciosos, com OR 5,38.

A duração do maior episódio ou a carga diária dos EAFAs que elevam o risco de eventos isquêmicos suficientemente para justificar a anticoagulação são incertas. A recomendação atual é seguir o algoritmo de gerenciamento dos EAFAs7, visto que não existem estudos publicados até o momento sobre esse assunto.

### Limitações

O estudo apresentado tem limitações relacionadas ao pequeno número de pacientes incluídos e ao seu desenvolvimento em um centro único de observação, mas vale ressaltar que não existe qualquer estudo sendo realizado com a população chagásica e com este objetivo. Além disso, diferentemente de outros estudos já realizados, foram incluídos aqui as três marcas de DCEIs mais utilizadas pela população estudada, não se limitando a um único tipo de dispositivo. A avaliação destes (programação realizada e análise dos registros) não interferiu em demais configurações, atendendo, portanto, às necessidades dos pacientes durante todo o tempo de monitoramento, e sendo o estudo facilmente reprodutível.

Ausência de um grupo controle também corresponde a uma limitação; contudo, mesmo sem o grupo controle, vale frisar a contribuição relevante do estudo para obtenção de informações inéditas sobre a doença de Chagas e os eventos isquêmicos.

A investigação dos eventos isquêmicos foi realizada por meio de TC de crânio. A sensibilidade para a detecção da isquemia é maior a partir de ressonância magnética (RM) de crânio. Entretanto, a utilização de RM específica para pacientes em uso de DCEIs ainda não é uma realidade para pacientes atendidos pelo sistema de saúde público brasileiro, e não foram realizadas análises de variabilidade intra e interobservador entre os médicos neurologistas.

## Conclusão

Observamos que em pacientes com doença de Chagas e DCEIs, os EAFAs ≥ 6 minutos são frequentes e sua associação com eventos isquêmicos silenciosos foi significante. A ocorrência de eventos isquêmicos silenciosos também se associou a uma maior carga diária máxima. Essa associação foi mais prevalente em pacientes idosos, e as demais características da doença de Chagas não interferiram nos resultados avaliados.

Estes são os primeiros resultados em pacientes chagásicos já publicados, e podem oferecer subsídios para profissionais que realizam rotineiramente o acompanhamento desses pacientes, tornando-os conscientes da relevância desses episódios e direcionando-os na busca e na aplicação de algoritmos para essa população específica.
