# Supplementary material for: Episódios de Alta Frequência Atrial e sua Associação com Eventos Isquêmicos Cerebrais em Pacientes Chagásicos
Source: Arq Bras Cardiol. 2020 Dec 1;115(6):1072–9. [Article in Portuguese] doi: 10.36660/abc.20190647 (PMC8133741; doi:10.36660/abc.20190647)
Supplement: Translation

# Translation: Atrial High-Rate Episodes and Their Association with Cerebral Ischemic Events in Chagasic Patients

**Authors:** Emanoela Lima Freitas, Elieusa e Silva Sampaio, Márcia Maria Carneiro Oliveira, Lucas Hollanda Oliveira, Marcos Sergio da Silva Guimarães, Jussara de Oliveira Pinheiro, Luís Pereira de Magalhães, Guisela Steffen Bonadie Albuquerque, Cristiano Macedo, Roque Aras

**Affiliations:** 1 Brazil Hospital Cardio Pulmonar, Salvador, BA - Brazil; 2 Brazil Universidade Federal da Bahia – Enfermagem, Salvador, BA - Brazil; 3 Brazil Escola de Enfermagem, Salvador, BA - Brazil; 4 Brazil Hospital Ana Nery, Salvador, BA - Brazil; 5 Brazil Hospital Universitário Professor Edgard Santos, Salvador, BA - Brazil; 6 Brazil Universidade Federal da Bahia Faculdade de Medicina de Bahia, Salvador, BA – Brazil

**Keywords:** Chagas Disease/complications, Atrial Flutter, Cerebral Infarction, Brain Ischemia, Pacemaker Artificial, Tomography, Computed/methods

## Abstract

**Background:**

Atrial high-rate episodes (AHREs) are associated with an increased risk of cerebral ischemic events; however, there are no studies related to the presence of AHREs and cerebral ischemic events in Chagasic patients.

**Objective:**

To investigate the association between the presence of AHREs ≥ 6 minutes and cerebral ischemic events in Chagasic patients.

**Methods:**

Cohort study with Chagasic patients with implantable electronic cardiac devices (IECDs), followed at the Arrhythmia Outpatient Clinic of a University Hospital, in the city of Salvador, state of Bahia, Brazil, between May 2016 and June 2017.. Patients diagnosed with atrial flutter / atrial fibrillation, with unicameral IECD and using oral anticoagulation were excluded. AHREs with atrial frequency ≥ 190 beats per minute and duration ≥ 6 minutes (min) were considered, and cerebral ischemic events were identified by computed tomography (CT) of the skull.

**Results:**

The 67 research participants (67.2% females, mean age 63.6 ± 9.2 years) were followed for 98 ± 28.8 days and 11.9% of the patients had AHREs ≥ 6 min. Skull CT showed silent cerebral ischemic events in 16.4% of the patients, 63.6% of whom had AHREs ≥ 6 min in the analysis of IECDs. Advanced age [OR 1.12 (95% CI 1.03-1.21; p=0.009] and the presence of AHREs ≥ 6 minutes [OR 96.2 (95% CI 9.4-987.4; p <0.001)] were independent predictors for ischemic events.

**Conclusion:**

AHREs detected by IECDs were associated with the presence of silent cerebral ischemic events in Chagasic patients. (Arq Bras Cardiol. 2020; [online].ahead print, PP.0-0)

## Introduction

Atrial fibrillation (AF) increases the risk of ischemic stroke by five to six times regardless of other risk factors. ^1^ In recent years, interest in detecting AF at an earlier stage, before clinical identification, has been growing, mainly detected through an implantable cardiac pacemaker / defibrillator (ICD) and preceding the first disease manifestation, ^2 , 3^ the atrial high-frequency episodes (AHREs), which correspond to the occurrence of atrial arrhythmias such as atrial fibrillation and flutter and are characterized by having an atrial frequency ≥ 190 beats per minute (bpm) ^4^ or ≥ 250 bpm, ^3^ with duration ≥ 5 to 6 minutes (min). They are asymptomatic episodes and detected only through continuous monitoring and are also called “subclinical AF”. ^4^

AHREs are associated with an increased risk of stroke ^5^ and the tendency is that these episodes have the same adverse prognosis as clinical AF; however, the duration, frequency or exact daily load of these episodes at risk of stroke is still unknown; thus, the threshold of AHREs that justifies oral anticoagulation is not yet clear. ^6^

The incidence of AHREs is 30-70%, depending on the clinical profile of the population studied and the detection algorithms used in each study protocol. ^7^ When excluding patients with a history of AF and using oral anticoagulation, that number drops to around 30%. ^8 - 10^

However, in some specific populations who are vulnerable to thromboembolic complications, such as patients with Chagas’ disease (CD), there are no data related to the investigation of the presence of these episodes and their incidence.

Approximately 30% of CD patients develop cardiac changes, and more than 10% develop neurological changes. ^11^ Complications of heart disease are mainly due to arrhythmias and heart failure, responsible for more than 35% of deaths. ^12^ In CD, AF is the most frequent supraventricular arrhythmia, being found in 4 to 12% of cases, ^13^ being one of the main causes of cerebral embolic events, ^14^ and of which incidence affects 3% of the Chagasic population. ^15^

Investigating the presence of AHREs and their association with stroke in patients with CD may allow the inclusion of these patients to those using oral anticoagulants. The objective was to investigate the association between the presence of AHREs ≥ 6 minutes and cerebral ischemic events in Chagasic patients.

## Methods

### Study Design and Population

This is an observational, prospective cohort study. All 77 patients were included, of both genders, aged ≥ 18 years, followed at the arrhythmia outpatient clinic of a University Hospital, a referral in Cardiology, in the city of Salvador, state of Bahia, Brazil, between May 2016 and June 2017. The patients had Chagas’ disease and IECD’s (pacemaker, implantable cardioverter defibrillator or cardiac resynchronization therapy devices) capable of monitoring atrial activity. Patients diagnosed with atrial fibrillation / atrial flutter, with unicameral IECD, those with chronic indication for oral anticoagulation for any reason, or those with a contraindication to cranial tomography were excluded.

The research was performed in accordance with the principles of the Declaration of Helsinki and approved by the Research Ethics Committee of Hospital Universitário Prof. Edgard Santos UFBA-HUPES (under number: 1,426,885, on 26/02/2016). The consent form was obtained from all participants.

Data on gender, age, ethnicity, comorbidities, type and indication of IECD’s, IECD stimulation mode ‘ drug therapy and characterization of Chagas disease were collected, in addition to data related to chest radiography, transthoracic echocardiogram ((TTE) and long-term electrocardiogram - 24-hour Holter. In each patient, CD was classified according to the criteria of the Brazilian Consensus on Chagas Disease. ^14^ Data were collected through interviews with patients and from medical records.

The risk score used was CHA _2_ DS _2_ -VASc (C- Congestive heart failure (or Left ventricular systolic dysfunction) – 1 point, H- Hypertension – 1 point, A _2__**-**_ Age ≥75 years - 2 points, D - Diabetes Mellitus – 1 point, S _2_ -Prior Stroke or transient ischemic attack or thromboembolism- 2 points, V - Vascular disease (e.g., peripheral artery disease, myocardial infarction, aortic plaque) – 1 points, A - Age 65–74 years – 1 point and Sc **-** Sex category (female sex) - 1 point). ^16^

The risk classification of cerebrovascular events, according to the score CHA2DS2-VASc is defined as follows: High risk (2 points or more), intermediate risk (1 point) and low risk (0 points).

### Study Procedures

After signing the informed consent form, patients underwent a 12-lead electrocardiogram (ECG), aiming at confirming the absence of atrial fibrillation / atrial flutter, in addition to identifying cardiac rhythm and intraventricular conduction disorders. Then, the patients were evaluated by an arrhythmologist and had their IECDs adjusted to a specific schedule, aiming at detection and recording of atrial arrhythmias.

After a period of approximately 3 months after the schedule implementation, the patients returned to the clinic to have devices analyzed (reading of the IECD’s), to identify and classify the occurrence of atrial arrhythmias, the AHREs.

During the period between the programming and the reading of the IECDs, patients underwent a non-contrast-enhanced skull computed tomography (CT) aiming to identify cerebral ischemic events. Silent cerebral infarction was identified in those patients who showed changes in cerebral infarction in the CT reports and who did not show any clinical changes in ischemic events or neurological deficits. CT scans were performed and evaluated by the Neuroradiology Department of the Hospital. This examination was performed on a Toshiba Medical Systems Corporation device, 1385 (Shimo Ishigami, Otawara-Shi, Tochigi, Japan).

### IECDs Programming

The choice of device manufacturer did not influence patient inclusion / exclusion. Manufacturers’ devices that have been included were Medtronic®, St. Jude Medical® and Biotronik®, of which models were available for use in this population.

The device was programmed to identify AHREs lasting at least 190 bpm, for ≥ 6 min, recognized as an appropriate cutoff point to select AHREs and rule out premature atrial contraction or false events, ^17^ throughout the monitoring period. This duration was chosen because it is consistent with the methodology of two large studies, the “Asymptomatic Atrial Fibrillation and Stroke Evaluation in Pacemaker Patients and the Atrial Fibrillation Reduction Atrial Pacing Trial (ASSERT)” ^4^ and the “The Relationship Between Daily Atrial Tachyarrhythmia Burden From Implantable Device Diagnostics and Stroke Risk (TRENDS)”, ^3^ which demonstrated the association between AHREs, lasting at least five or six min, and cerebral ischemic events. The storage electrogram was activated to confirm the occurrence of AHREs. Patients had atrial sensitivity set at 0.1 - 0.5 millivolts (mV).

All AHREs detected by IECD lasting ≥ 6 min and with frequency ≥ 190 bpm were documented and sent for blind assessment by the specialist (electrophysiologist). The activation of other electrogram storage triggers was left to the physician’s discretion but was not supported by the study protocol.

In addition to device programming, data related to stimulation / detection parameters, percentages of atrial and ventricular utilization, as well as the minimum and maximum heart rate were collected.

### Monitoring

Approximately three months after IECD programming, the patient returned for the subsequent consultation, and at this time data related to the detection of AHREs were collected.

The atrial electrograms corresponding to the detected AHREs were evaluated by an electrophysiologist blinded to the results of cranial CT scans. These electrograms were categorized as adequate or inadequate detections. Inadequate detections (noise, ventricular Far-field R wave detection, or repetitive, non-reentrant ventricular-atrial synchrony), ^17 , 18^ were excluded.

Three categories were defined, without overlapping the duration of AHREs: (1) without AHREs; (2) AHREs lasting <6 min; (3) AHREs lasting ≥ 6 min. Based on the detection of AHREs lasting ≥ 6 min, 4 subgroups were defined: (1) AHREs between 6 min - 29 min; (2) AHREs between 30min - 5h59min; (3) AHREs between 6 am - 11:59 pm and (4) AHREs ≥ 24 h. These cutoffs were defined according to the positive predictive values established by the ASSERT study analysis. ^19^

For AHREs ≥ 6 minutes and ≥ 190 beats / min, the positive predictive value was 82.7%. The positive predictive value increased to 93.2%, 96.7% and 98.2% when the threshold duration was extended to 30 minutes, six hours and 24 hours, respectively. Increasing the threshold heart rate to 250 beats / min decreased false positive detections, but to a lesser extent, and added only marginally to the positive predictive value when long threshold durations were used. ^19^

### Ischemic Events in Cranial Tomography

All CT scans were performed and evaluated by the Department of Neuroradiology of the Hospital where the study was conducted, with the aim of identifying areas compatible with ischemic events (ischemic areas, lacunar infarctions, localized glioses or hypodense areas). For patients with no medical history or previous neurological deficits, ischemic events were considered to be silent. The cranial CTs were performed on a Toshiba Medical Systems Corporation device, 1385, Shimoishigami, Otawara-Shi, Tochigu, Japan.

### Statistical Analysis

The statistical analysis was performed using the IBM SPSS program, version 21.0 (IBM, Armonk, New York). The data was submitted to descriptive statistical analysis, using frequency measurements (absolute and relative) for qualitative variables. For quantitative variables, mean and standard deviation, or median and interquartile range were used, depending on the variable distribution, which was tested using the Shapiro-Wilk test.

For the categorical variables, the χ ^2^ test or Fisher’s exact test was applied. The unpaired Student’s t test was used for the mean ages of patients with AHREs ≥ 6min and without AHREs ≥ 6min and for the comparison of LVEF means, whereas the Mann-Whitney test was used to compare the time since the IECD implantation in months.

The results were presented using Odds Ratio (OR) and their respective 95% confidence intervals (95%CI). A value of p <0.05 was considered statistically significant.

## Results

### Population Characteristics and Detection of AHREs

A total of 77 patients were included in the cohort, of which ten were excluded; seven had AF at the time of the cardiac device programming , and oral anticoagulation was started (as defined by the patient’s attending physician); two died before the reading of the device and in one patient, ventricular Far-field R-wave detection was identified during IECD reading. In the end, 67 participants completed all stages of the research.

The mean age was 63.6 ± 9.2 years; all participants were in the chronic phase of CD, 89.6% of whom had developed the Cardiac form and 10.4% the Cardiodigestive form of the disease. The clinical manifestations of the cardiac form of CD included the Arrhythmic Syndrome (100% of patients), Heart Failure - HF (38.8%) and Thromboembolic Syndrome (3% - corresponding to two patients with previous stroke). The clinical manifestations related to the Cardiodigestive form was the occurrence of Chagasic Megaesophagus in seven patients.

As for data related to IECDs, we found that 46.3% of patients were in DDD stimulation mode (double-chamber stimulation), followed by 41.8% in DDD-R mode (DDD with frequency response - R); the minimum heart rate was 61.8 ± 3.9 bpm, and the maximum was 124.4 ± 5.5 bpm. The percentage of atrial utilization averaged 52.8 ± 37.4%, whereas the ventricular was 65.6 ± 42.5%.

The mean follow-up was 98 ± 28.8 days and AHREs were detected in 24 (35.8%) patients, with varying durations. The incidence of AHREs lasting ≥ 6 minutes or “subclinical AF” was 11.9% (n = 08).

The median time to reach the first AHRE was 26.2 days (ranging from 0.08 to 83.25 days), and the median duration of the AHREs was 135.4 minutes (ranging from 22.8 to 5811.8 minutes).

Comparisons of the demographic and clinical characteristics of patients with AHREs ≥ 6min *versus* patients without AHREs or with duration <6 min are shown in table 1 .

**Table 1:** – Demographic and clinical characteristics of patients with AHREs ≥ 6 min *versus* patients without AHREs or duration <6 min

Demographic and clinical characteristics	Population total (n=67)	With AHREs ≥ 6min (n= 08)	Without AHREs or < 6min (n= 59)	P value
Age (years) – mean ± SD	63.6 ± 9.2	69.9 ± 10.4	62.8 ± 8.8	0.040 ^†^
Sex– n (%)				0.103
Female	45 (67.2)	3 (6.7)	42 (93.3)	
Male	22 (32.8)	5 (22.7)	17 (77.3)	
Ethnicity – n (%)				1.000
White	4 (6)	0(0.0)	4 (100)	
Nonwhite	63 (94)	8 (12.7)	55 (87.3)	
IECD Type				1.000
Pacemaker	62 (92.5)	8 (12.9)	54 (87.1)	
ICD	5 (7.5)	0(0.0)	5 (100)	
IECD Indication – n (%)				1.000
AVB/ TAVB	52 (77.6)	7 (13.5)	45 (86.5)	
SND	10 (14.9)	1 (10.0)	9 (90)	
Secondary prevention SCD	5 (7.5)	0(0.0)	5 (100)	
Implant OF IECD (months) ^§^	108 (48-168)	144 (54-165)	96 (36-180)	0.757*
HF (NYHA)	26 (38.8)	4 (15.4)	22 (84.6)	0.701
HF functional class				1.000
I	7 (26.9)	1 (14.3)	6 (85.7)	
II	13 (50)	2 (15.4)	11 (84.5)	
III	6 (23.1)	1 (16.7)	5 (83.3)	
AMI– n (%)	4 (6)	8 (12.7)	4 (100)	1.000
SAH – n (%)	50 (74.6)	6 (12)	44 (88)	1.000
Diabetes – n (%)	6 (9)	1 (16.7)	5 (83.3)	0.549
Dyslipidemia – n (%)	21 (31.3)	2 (9.5)	19 (90.5)	1.000
LVEF % - mean ± SD	58.5 ± 14.1	58.1 ± 11	58.6 ± 14.5	0.495
LA ≥ 40mm – n (%)	22 (32.8)	3 (13.6)	19 (86.4)	1.000
CHA2DS2-VASc Score				0.346
Low risk	12 (17.9)	1 (8.3)	11 (91.7)	
Intermediate risk	37 (55.2)	3 (8.1)	34 (91.9)	
High risk	18 (26.9)	4 (22.2)	14 (77.9)	

Source: The author. Data is presented as mean ± standard deviation or the number of patients (%); § Data presented as median and interquartile range. P values were calculated using the Chi-square test, * Mann-Whitney and the † Student’s t test, as appropriate. IECD: Implantable electronic cardiac device. ICD: Implantable cardioverter defibrillator. AVB/TAVB: Atrioventricular block / Total atrioventricular block. SND: Sinus node disease. SCD: Sudden cardiac death. HF: Heart Failure (NYHA: New York Heart Association). AMI: Acute myocardial infarction. SAH: Systemic arterial hypertension. LVEF: Left ventricular ejection fraction. LA: Left atrium. CHA2DS2-VASc: risk score for thromboembolic events (C- Congestive heart failure (or Left ventricular systolic dysfunction) – 1 point, H- Hypertension – 1 point, A _2_ - Age ≥75 years- 2 points, D - Diabetes Mellitus – 1 point, S _2_ - Prior Stroke or transient ischemic attack or thromboembolism- 2 points, V - Vascular disease (e.g. peripheral artery disease, myocardial infarction, aortic plaque) – 1 point, A - Age 65–74 years – 1 point e Sc - Sex category (female sex) - 1 point).

### Detection of Ischemic Events

Eleven patients (16.4%) had an ischemic event on cranial CT and had no history of previous stroke. It was observed that 87.5% of the patients, who had AHREs ≥ 6 min, also had ischemic events on cranial CT. Table 2 shows the clinical characteristics of patients with and without ischemic events.

**Table 2:** – Clinical characteristics of patients with and without ischemic events

Clinical characteristics	Ischemic events (n= 11)	Non-ischemic events (n= 56)	P value
Age (years) – mean ± SD	70.6 ± 10.9	62.2 ± 8.3	0.005†
Sex– n (%)			0.483
Female	6 (13.3)	39 (86.7)	
Male	5 (22.7)	17 (77.3)	
HF	6 (23.1)	20 (76.9)	0.315
Classification of HF -(NYHA)			0.843
I	1 (14.3)	6 (85.7)	
II	3 (23.1)	10 (76.9)	
III	2 (33.3)	4 (66.7)	
AMI – n (%)	1 (25)	3 (75)	0.521
SAH – n (%)	8 (16)	42 (84)	1.000
DM – n (%)	1 (16.7)	5 (83.3)	1.000
Dyslipidemia – n (%)	5 (23.8)	16 (76.2)	0.301
LVEF % - mean ± SD	57.2 ± 14	58.7 ± 14.2	0.553†
LA ≥ 40mm – n (%)	6 (27.3)	16 (72.7)	0.157
Score CHA2DS2-VASc			0.050
Low risk	-	12 (100)	
Intermediate risk	5 (13.5)	32 (86.5)	
High risk	6 (33.3)	12 (66.7)	
AHREs			< 0.001
Without AHREs	3 (7)	40 (93)	
AHREs < 6minutes	1 (6.3)	15 (93.8)	
AHREs ≥ 6minutes	7 (87.5)	1 (12.5)	

*Source: The author. Data is presented as mean ± standard deviation or the number of patients (%); § Data presented as median and interquartile range. P-values were calculated using the Chi-square test and † Student’s t test. AHREs: Atrial high-rate episodes. IECD: Implantable electronic cardiac device. PM: Cardiac pacemaker. AVB/TAVB: Atrioventricular block / Total atrioventricular block. SND: Sinus node disease. SCD: Sudden cardiac death. HF: Heart Failure (NYHA: New York Heart Association). AMI: Acute myocardial infarction. SAH: Systemic arterial hypertension. DM: Diabetes mellitus. LVEF: Left ventricular ejection fraction. LA: Left atrium. CHA2DS2-VASc: risk score for thromboembolic events (C- Congestive heart failure (or Left ventricular systolic dysfunction) – 1 point, H- Hypertension – 1 point, A _2_ - Age ≥75 years- 2 points, D - Diabetes Mellitus – 1 point, S _2_ - Prior Stroke or transient ischemic attack or thromboembolism- 2 points, V - Vascular disease (e.g. peripheral artery disease, myocardial infarction, aortic plaque) – 1 point, A - Age 65–74 years – 1 point e Sc - Sex category (female sex) - 1 point).*

It was observed that 45.5% of the patients with ischemic events had AHREs lasting between 30min and 05h59 min, and the average number of AHREs ≥ 6 min was 3.88 ± 2.58, with 50% of patients having between 1 and 3 episodes.

In addition to considering the longest AHRE identified by the IECD, the total daily load of AHREs (the maximum time that the patient remained in “subclinical AF” during a 24-hour period) and its possible association with ischemic events were also measured. It was demonstrated that the Chagasic patient with daily load lasting ≥ 06 minutes, is more likely to develop ischemic events [OR: 46.67 (6.57 - 331.67; p <0.001)]. The median maximum daily load that was associated with the occurrence of ischemic events was 4,554 seconds (75.9 minutes) [OR: 1.001; p <0.026).

Table 3 shows the description of the daily load of AHREs in patients with and without ischemic events.

**Table 3:** – Description of the daily load of AHREs in patients with and without ischemic events

Description of daily load	With ischemic events (n= 11)	No ischemic events (n= 56)	P value
Daily load of AHREs - n (%)			< 0.001
Without daily load	3 (27.3)	40 (71.4)	
< 6 minutes	1 (9.1)	14 (25)	
≥ 6 minutes	7 (63.6)	2 (3.6)	

*Data is presented as the number of patients (%); The P-value was calculated using Fischer’s exact test. AHREs: Atrial High Rate episodes.*

Advanced age and the presence of AHREs ≥ 6 minutes were associated with ischemic events, as shown in Table 4 .

**Table 4:** – Factors associated with ischemic events

Associated factors	OR	CI 95%	P value
AHREs ≥ 6 minutes	96.2	9.4 - 987.5	< 0.001
Advanced age	1.12	1.03 - 1.21	0.009
HF	2.16	0.58 -7.98	0.241
Dyslipidemia	2.08	0.56 – 7.80	0.270
SAH	0.89	0.21 -3.82	0.874

*OR: Odds Ratio; 95%CI: 95% confidence interval; χ2 test. AHREs: Atrial high-rate episodes. HF: Heart Failure. SAH: Systemic arterial hypertension.*

## Discussion

There was an association of AHREs ≥ 6 min with silent ischemic events. The study by Benezet-Mazuecos et al. ^20^ showed that silent cerebral ischemic events occur more in patients with AHREs (42%) than in those without AHREs (19%), finding an OR of 3.4. Benezet-Mazuecos et al. ^21^ also described that silent cerebral ischemic events occurred more in patients with AHREs (32%) than in those without AHREs (13%) finding an OR of 2.45. In addition, brief episodes of subclinical AF (48 hours) documented by Holter monitoring were associated with a significantly increased risk of silent cerebral ischemic events and stroke. ^22^

The incidence of AHREs in patients without a history of AF is around 30%, with different monitoring periods. ^4 , 9 , 10^ In our study, the incidence found in the Chagas population (also without a history of AF), with a monitoring period of about 03 months, was 11.9%. This finding is very similar to that found in a large study of 2,580 non-Chagasic patients with no history of AF, where AHREs ≥ 6 min were found in 35% of patients in an average follow-up of 2.5 years, and in 10% of patients in the first three months of the study. ^4^ However, it is important to note that the median time to detect episodes in our study with Chagasic patients (26.2 days) was lower than the one in other studies, as in the MOde Selection Trial (MOST) - 100 days ^2^ and ASSERT - 36 days, ^4^ and additionally, the average number of AHREs in our study was higher (3.9) than that in the ASSERT study (2.0). ^4^

In this study, all patients had a double-chamber IECD; however, no association was found between the dual-chamber stimulation mode and the presence or absence of AHREs, and this may be due to the fact that many episodes were short-lived. However, it is possible that in a larger sample of patients, a reduction in AHREs can be detected, with double-chamber stimulation. The MOST ^2^ study also did not show this association.

The detection of AHREs was higher in patients with advanced age, females, of black ethnicity, with a history of AMI, whose indication for IECD implantation was AVB / TAVB; however, only advanced age was associated with the ischemic event. The aging of the population results in an increase in the underlying heart disease, and the improvement in diagnostic techniques has shown a high prevalence of AF in people over 70 years of age. ^23^ This also happens with the Chagasic population, ^24^ where the prevalence of AF was markedly higher in individuals with advanced age. Studies have shown that AHREs were more prevalent in the elderly population, but no statistical significance was found in this association. ^4 , 20^

In addition to the silent ischemic events associated with AHREs ≥ 6 min in older patients, there was also an association of the high-risk CHA2DS2-VASC score with silent ischemic events, similar to what was observed in other studies. ^20 , 21 , 25^

Although AHREs detected by IECDs are associated with a 2- to 2.5-fold increase in stroke risk, compared to individuals without AHREs, ^3 , 4^ the absolute risk of stroke in these patients is lower than in those with detected clinical AF. ^26^

Detection studies of “subclinical AF” using IECDs have tried to identify the ideal time limit (considering the longest episode or daily load) and its clinical consequences, such as thromboembolic events. The duration of the thresholds described in the studies have been highly variable, 5 min, ^2 , 3^ suggesting an increase of 2.8 in the risk of stroke or death; 6 min, ^4^ with a 2.5-fold increased risk for thromboembolism; 24 h ^8^ with an increase of 3.1, with a greater risk for thromboembolic events. Likewise, the daily load of 3.8 ^27^ and 5.5 h, ^3^ has also been associated with a significant increase in stroke risk (9 and 2-fold increase, respectively).

In our study, the daily load of AHREs lasting ≥ 6 minutes, suggests a greater chance for the Chagasic patient to develop a silent ischemic event, a higher risk than that described by other studies. In patients with IECDs, ^28^ an OR of 2.11 was demonstrated for the occurrence of ischemic events in the patient who had at least 1 day with at least 1 hour of the “subclinical AF” load (95% CI: 1.22-3.64, p = 0.008). Another study, ^20^ describes that the load detected by the IECDs was shown to be significantly associated with silent cerebral ischemic events, with an OR o 5.38.

The duration of the longest episode or the daily load of AHREs that sufficiently increases the risk of ischemic events to justify anticoagulation is uncertain. The current recommendation is to follow the AHRE Management Algorithm ^7^ , since there are no studies published so far on this subject.

### Limitations

The present study has limitations related to the small number of patients included in it and its development in a single observation center, but it is worth mentioning that there are no studies being carried out with the Chagasic population and with this purpose. In addition, unlike other previously performed studies, the three brands of IECDs most often used by the studied population were included here, not limited to a single type of device and their evaluation (programming performed and analysis of records) did not interfere with other configurations, therefore meeting the needs of patients during the entire monitoring time, with the study being easily reproducible.

The absence of a control group also corresponds to a limitation; however, even without the control group, it is worth emphasizing the relevant contribution of the study to obtain unprecedented information on patients with Chagas disease and ischemic events.

The investigation of ischemic events was performed through skull CT, and the sensitivity for the detection of ischemia is greater than that of skull magnetic resonance. However, the use of specific magnetic resonance imaging methods for patients using IECDs is not yet a reality for patients treated by the Brazilian public health system and analyses of intra and inter-observer variability have not been carried out by neurologists.

## Conclusions

We observed that in patients with Chagas disease and IECDs, AHREs ≥ 6 min are frequent and their association with silent ischemic events was significant. The occurrence of silent ischemic events was also associated with a higher maximum daily load. This association was more prevalent in elderly patients, and the other characteristics of Chagas disease did not interfere with the evaluated results.

These are the first published results about Chagasic patients, and may offer subsidies for professionals who routinely monitor these patients, making them aware of the relevance of these episodes and directing them in the search and application of algorithms for this specific population.
